# Clinical Characteristics and Treatment Outcomes of Acute Myeloid Leukemia in Adolescent and Young Adult versus Adult Patients: A Single-Center Experience in Qatar

**DOI:** 10.46989/001c.140933

**Published:** 2025-06-23

**Authors:** Shehab Fareed, Abdulrahman Fadhl Al-Mashdali, Hawraa Shwaylia, Yahya Mulikandathil, Awni Alshurafa, Sarah Aldali, Deena Sideeg Mudawi, Dina Soliman, Feryal Abbas, Mohammed Abdulgayoom, Anil Yousef, Kaplana Singh, Honar Cherif, Mohamed Yassin

**Affiliations:** 1 Division of Hematology, Department of Medical Oncology, National Center for Cancer Care & Research (NCCCR), Hamad Medical Corporation (HMC), Doha, Qatar; 2 Department of Pharmacy, National Center for Cancer Care and Research, Hamad Medical Corporation, Doha, Qatar; 3 Department of Laboratory Medicine and Pathology, National Center for Cancer Care and Research, Hamad Medical Corporation, Doha, Qatar; 4 Nursing Research Department, Hamad Medical Corporation, Doha, Qatar

**Keywords:** Acute Myeloid Leukemia (AML), Adolescents and Young Adults (AYA), AML Management, Age-Related Differences, Hematology

## Abstract

Acute myeloid leukemia (AML) presents differently across age groups, with unique challenges in the adolescent and young adult (AYA) populations. This study compares clinical characteristics and outcomes between AYA and adult AML patients in Qatar. We conducted a retrospective analysis of 151 AML patients treated at the National Center for Cancer Care and Research, Qatar, between 2017-2021. Patients were divided into AYA (15-39 years, n=73) and adults (≥40 years, n=78) groups. Clinical characteristics, cytogenetic profiles, treatment approaches and survival outcomes were compared between groups. AYA patients (median age 30 years) showed distinct characteristics compared to adults (median age 53.9 years). AYA patients had lower platelet counts (62,900/mm³ versus 96,500/mm³, p=0.016) and higher blast percentages in peripheral blood and bone marrow (60% versus 40%, p=0.02). Core binding factor rearrangements were more common in AYA patients (32% versus 12%, p=0.03), while adult patients had more diploid karyotypes (55% versus 36%). AYA patients received more intensive therapy, with higher rates of FLAG-Ida salvage therapy (34% versus 15%) and allogeneic transplantation (32% versus 15%, p=0.01). While transplantation significantly improved survival in adults, its impact was less pronounced in AYA patients. Median overall survival was comparable between groups (23.14 versus 24.08 months, p=0.09). Our study reveals distinct biological and clinical characteristics between AYA and adult AML patients in Qatar. Despite receiving more intensive therapy, AYA patients showed comparable survival outcomes to adults, suggesting the need for age-specific treatment approaches. The differential impact of transplantation between age groups highlights the importance of personalized treatment strategies. These findings contribute to understanding age-specific differences in AML and may help optimize treatment approaches for both populations.

## Introduction

Acute myeloid leukemia (AML) is an aggressive hematologic malignancy characterized by the clonal proliferation of immature myeloid cells in the bone marrow, blood and other tissues. This uncontrolled growth impairs normal hematopoiesis, leading to symptoms such as anemia, infection, and bleeding. AML is the most common acute leukemia in adults, with an incidence increasing with age. The prognosis and treatment approaches vary based on cytogenetic and molecular profiles.^1,2^ AML is predominantly a disease of older adults, with a typical diagnosis age between 68 and 72 years. However, adolescents and young adults (AYA), aged 15 to 39 years as defined by NCCN guidelines, also present with AML, although the information available for this age group is limited and heterogeneous.^3^

Age-related differences in genetic and molecular profiles significantly influence disease characteristics across different age groups. Molecular studies have demonstrated that certain genetic mutations become more prevalent as patients age. AYA patients typically present with distinct genetic profiles, often showing a higher frequency of normal cytogenetics compared to older populations. Beyond the biological aspects, younger patients face unique challenges that extend well beyond their medical condition. These individuals must navigate their illness while simultaneously managing crucial life milestones, including educational pursuits, career development, establishing independence, and family planning. These concurrent life challenges can add substantial psychological and social burden to their disease experience.^4^ Unlike AYA patients with acute lymphoblastic leukemia (ALL), who tend to have a more favorable prognosis, those with AML often face a significantly poorer outlook despite advancements in treatment options This is partly due to most clinical trials and therapeutic protocols having been developed based on pediatric studies, with fewer focusing on the adult population.^5^

This retrospective study aims to shed light on the clinical features, cytogenetic abnormalities, molecular prognostic markers, treatment approaches and outcomes of AML in AYA patients compared with adult population, providing a detailed analysis of this less frequently studied group.

## Methods

We conducted a retrospective comparative analysis using data from the clinical database of our single tertiary hematology center, The National Center for Cancer Care and Research, Doha, Qatar. This center serves a country’s population of approximately 2.8 million people with an estimated median age of 32.2 years. The study included all patients diagnosed with AML between January 2017 and December 2021. They were stratified into two groups: adolescents and young adults (AYA, age 15-39 years) and adults (age ≥40 years).

For both age groups, we collected comprehensive data including demographic information, clinical characteristics, laboratory findings (hematological profiles), pathological features, and molecular attributes. Data exhibiting normal distribution were represented as means with standard deviations (SD) and corresponding 95% confidence intervals (CI). Non-normally distributed data were summarized using median values and interquartile ranges (IQR). Categorical variables were expressed as frequencies and percentages.

The study aimed to compare clinicopathological features between AYA and adult patients with AML, focusing on:

- Demographic and clinical presentation
- Morphological characteristics
- Immunophenotypic profiles
- Cytogenetic alterations
- Molecular features
- Treatment approaches and outcomes

Statistical comparisons between the two age groups were performed using Student’s t-test or Mann-Whitney U test for continuous variables, and chi-square or Fisher’s exact test for categorical variables. Survival metrics, including overall survival (OS) and progression-free survival (PFS), were computed using the Kaplan-Meier method. OS was calculated from the time of diagnosis in newly diagnosed patients or from the treatment start date in relapsed disease to the time of death or last follow-up. The log-rank test was used to compare survival curves between age groups and subgroups. All statistical analyses were performed using GraphPad Prism version 9.0.0, with p-values <0.05 considered statistically significant.

## Results

### Patient Demographics and Disease Characteristics

Among the 151 AML patients treated at our center between 2017 and 2021, we identified 73 adolescents and young adults (AYA) and 78 adults, representing 48.3% and 51.7% of the cohort, respectively. The median age differed significantly between groups (30 years for AYA versus 53.9 years for adults, p<0.001). Both groups showed similar male predominance (75% in AYA, 78% in older adults). A notable demographic distinction was observed in ethnic distribution, with Asian descent predominating in the AYA group (63%) compared to only 33% in the adult group, where Arab ethnicity was more prevalent. Patient characteristics are described in Table 1.

**Table 1.** General Characteristics of Patients

	**Number/%/Status**	Number (percentage)
**Total number**	73	
**Age, mean (SD)**	30.0 (6.3)	
**Gender**		
Male	55 (75%)	
Female	18 (25%)	
**Ethnicity**		
Asian	46 (63%)	
Arab	22 (30%)	
Others	5 (7%)	
**HB, mean (SD)**	7.8 (2.8)	
**WBC, mean (SD)**	54,600/mm³ (80.8)	
**Platelet, mean (SD)**	62,900/mm³ (61.6)	
**Type of AML**		
De novo	66 (90%)	
Secondary	7 (10%)	
**Peripheral Blast, mean (SD)**	0.6 (0.3)	
**BM Blasts, mean (SD)**	0.7 (0.2)	
**Cellularity**		
Hypercellular	57 (86%)	
Normocellular	4 (6%)	
Hypocellular	5 (8%)	
**Dyserythropoiesis**		
No	56 (77%)	
Yes	17 (23%)	
**Dysgranulopoiesis**		
No	44 (60%)	
Yes	29 (40%)	
**Dysmegakaryopoiesis**		
No	64 (88%)	
Yes	9 (12%)	
**Risk cytogenetics**		
Favorable	24 (35%)	
Intermediate	31 (46%)	
Poor	13 (19%)	
FLT-3	Negative	46 (78%)
	Positive	13 (22%)
NPM-1	Negative	49 (82%)
	Positive	10 (17%)
	Not available	1 (2%)
**Second line**	FLAG-Ida	24 (34%)
	Hypomethylating agent	1 (1%)
	Others	1 (1%)
**Transplant**	No	50 (68%)
	Yes	23 (31%)
**Survival**	Alive	59 (81%)
	Dead	14 (19%)

### Laboratory Parameters and Disease Features

Initial laboratory findings revealed distinct patterns between the age groups. AYA patients presented with lower hemoglobin levels (7.8 g/dL) compared to adults (8.7 g/dL), and significantly lower platelet counts (62,900/mm³ versus 96,500/mm³, p=0.016). The disease burden, as measured by blast percentages, was notably higher in AYA patients, with 60% in peripheral blood and 70% in bone marrow, compared to 40% and 60% respectively in adults (p=0.02). Bone marrow examination showed further distinctions, with high cellularity being more common in AYA patients (77%). Notably, hypocellularity was present in 8% of AYA patients but absent in adults (p=0.03). Among AYA patients, dysplastic features included dysgranulopoiesis (40%), dyserythropoiesis (23%), and dysmegakaryopoiesis (12%).

### Cytogenetic and Molecular Profiles

Significant differences emerged in cytogenetic patterns between the age groups, as detailed in Table 2. Adults showed a higher frequency of diploid karyotypes (55% versus 36% in AYA), while CBF rearrangements were more prevalent in AYA patients (32% versus 12% in adults, p=0.03). Risk classification in AYA patients revealed 35% favorable, 46% intermediate, and 19% high risk. Molecular analysis showed comparable rates of FLT3-ITD (22%) and NPM1 (17%) mutations between the groups.

**Table 2.** Comparison of Characteristics Between Adolescents and Young Adults (AYA) and Non-AYA Patients

**Parameter**	variable	AYA (No, %)	Non AYA No, %	P-value
**Number of patients**		73	78	
**Age, mean (SD)**		30.0 (6.3)	53.9 (10.7)	<0.001
**Gender**	Female	18 (25%)	17 (22%)	0.68
	Male	55 (75%)	61 (78%)	
**Race**	Asian	46 (63%)	36 (46%)	0.11
	Arabs	22 (30%)	33 (42%)	
	Others	5 (7%)	9 (12%)	
**HB, mean (SD)**		7.8 (2.8)	8.7 (2.9)	0.070
**WBC, mean (SD)**		54.6 (80.8)	36.2 (48.3)	0.089
**PLT, mean (SD)**		62.9 (61.6)	96.5 (100.2)	**0.016**
**Blast, mean (SD)**		60% (0.3)	40% (0.3)	**0.002**
**BM Blast, mean (SD)**		70% (0.2)	60% (0.2)	**0.024**
**Cellularity**	Hypercellular	57 (78%)	67 (86%)	**0.038**
	Normocellular	4 (65.4%)	10 (13%)	
	Hypocellular	5 (8%)	0 (0%)	
	No BM biopsy given	0 (0%)	1 (1%)	
**Dyserythropoiesis**	Yes	17 (23%)	9 (12%)	0.084
	No	56 (77%)	64 (88%)	
**Dysgranulopoiesis**	Yes	29 (40%)	29 (40%)	1.00
	No	44 (60%)	44 (60%)	
**Dysmegakaryopoiesis**	Yes	9 (12%)	13 (18%)	0.35
	No	64 (88%)	60 (82%)	
**Cytogenetics**				0.036
	CBF	23 (32%)	9 (12%)	
	MLL/KMT2Ar	8 (12%)	7 (9%)	
	Others	10 (14%)	15 (19%)	
	Complex karyotype	2 (3%)	4 (5%)	
	Diploid	26 (36%)	43 (55%)	
**FLT-3**	Negative	46 (78%)	59 (80%)	0.80
	Positive	13 (22%)	15 (20%)	
**NPM-1**	Negative	49 (83%)	63 (85%)	0.74
	Positive	10 (17%)	11 (15%)	
**Allo-SCT**	No	50 (68%)	66 (85%)	**0.019**
	Yes	23 (32%)	12 (15%)	

### Treatment Approaches and Response

Treatment strategies varied significantly between age groups. While both groups received standard anthracycline-cytarabine induction therapy, FLAG-Ida (Fludarabine, Ara-C, Granulocyte-colony stimulation factor, Idarubicin) salvage therapy was more frequently employed in AYA patients (34% versus 15% in adults). The rate of allogeneic transplantation was significantly higher in AYA patients (32% versus 15% in adults, p=0.01).

### Survival Outcomes

**Figure 1.**
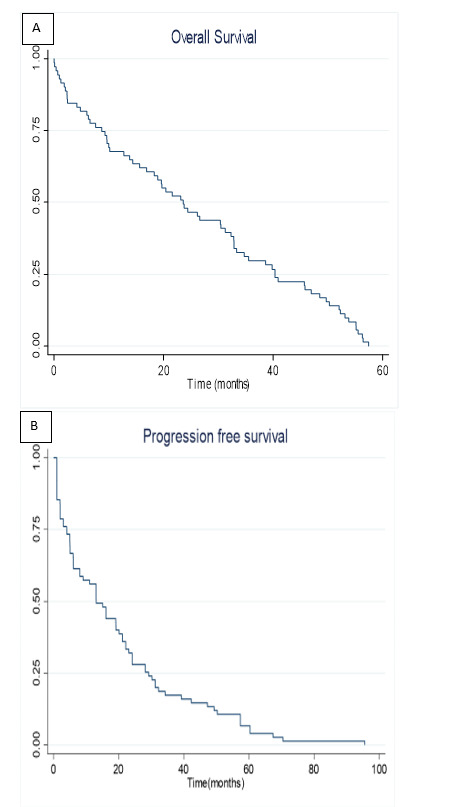
Overall (A) and Progression-free (B) survival curves in AYA patients

**Figure 2.**
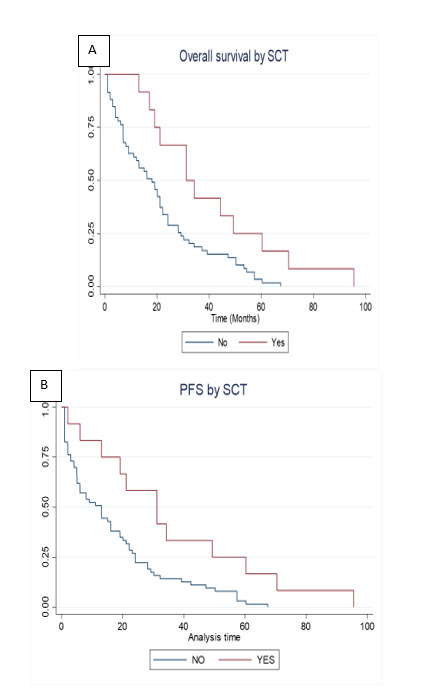
Overall survival(A) and progression-free survival (B) in non-AYA (adult)patients stratified by Stem Cell Transplantation Status (SCT versus No SCT; P=0.04)

**Figure 3.**
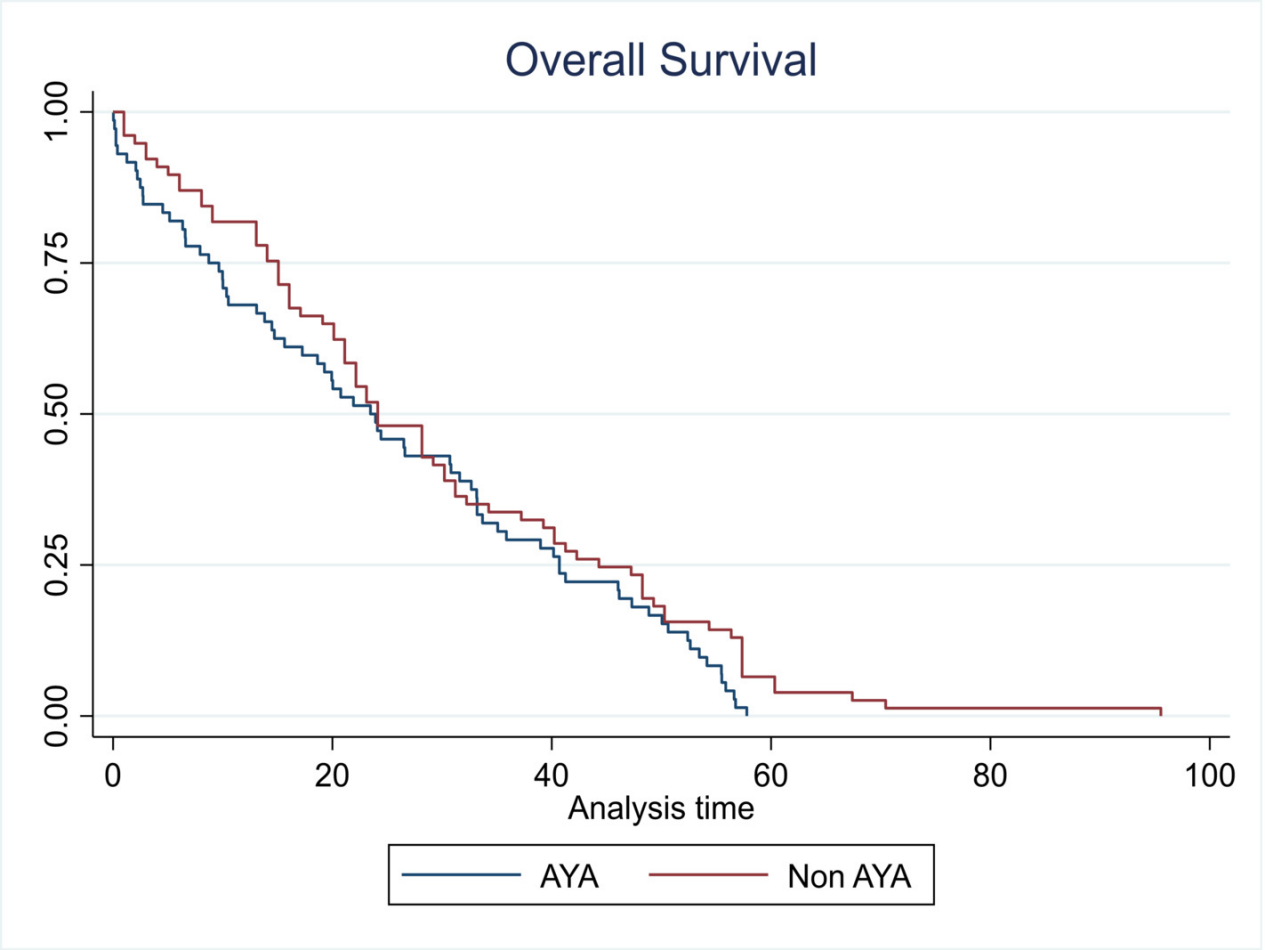
Median OS comparison between AYA and non-AYA(Adult) cohorts (P=0.09)

With a median follow-up of 24 months for the entire cohort, the survival analysis revealed interesting patterns. AYA patients demonstrated OS of 23.60 and PFS of 23.14 months (illustrated in Fig. 1 A, B). The impact of transplantation varied markedly between age groups. In adults, transplantation significantly improved both OS (24 versus 11 months) and PFS (31 versus 13 months) (p=0.04) (as depicted in Fig.2 A, B). However, AYA patients showed no significant survival difference with transplantation (OS: 26 versus 20 months, p=0.7; PFS: 23 versus 19 months, p=0.1). Overall comparison showed comparable median OS between AYA and adult patients (23.14 versus 24.08 months, p=0.09), as depicted in Fig. 3.

## Discussion

According to Qatar national registry (QNCR), the total population of Qatar reached 2,833,679 by 2020. The crude incidence of AML was found to be 5.08 per 100,000 and the age standardized rate (ASR) to be 7.68 per 100,000. The crude cumulative was 0.72. and the peak male incidence was 35-39.

AML is traditionally considered a disease of the elderly, with a higher incidence in older adults. However, in Qatar, the unique demographic structure with a predominantly working-age population results in a notable number of AML cases among AYA, typically defined as those between 15 and 39 years old. Our research compares the characteristics and outcomes between AYA patients and adults (age ≥40 years) with AML in Qatar, aiming to identify distinct patterns in disease biology, treatment responses, and long-term prognosis between these age groups.

Through this comparative analysis, we sought to understand age-specific differences that could influence treatment strategies. By examining these age-related variations, we aimed to develop tailored management recommendations that better suit the needs of each age group, ultimately improving treatment outcomes and survival rates for both AYA and adult patients.

AML accounts for 15%–20% of all leukemias in children and about 33% in adolescents and young adults. Age is a crucial prognostic factor in AML, with older age associated with a less favorable prognosis.^6,7^ Over the past two decades, the OS rate for children with AML has improved significantly, with a 5-year ranging from 60% to 75%. Despite these advances, the overall cure rates for AYA patients remain only 50%–60%.^8–11^ Differences in treatment protocols and clinical trial designs between pediatric and adult groups contribute to this discrepancy, as some patients receive differing treatment regimens.^12,13^

Data from the USA and Germany indicate variations in incidence and outcomes among AYA patients due to ethnic, racial, and socio-economic factors. Morphological and cytogenetic features may also differ in this age group.^14–16^ For instance, specific cytogenetic abnormalities, such as t (8;21), are less common in young adults compared to children, and AYA patients are more likely to present with normal cytogenetics and unfavorable genetic mutations like FLT3/ITD compared to younger children. However, AYA patients often have more favorable prognostic markers, such as CEBPA and NPM1 mutations.^2^

In our retrospective study of AML in AYA group, we observed a cytogenetic risk distribution of 35% favorable, 46% intermediate, and 19% high risk. These findings are generally consistent with global data on AML in the AYA population, where intermediate risk is often the most prevalent category.^17^ Our study found FLT3-ITD mutations in 22% of patients and NPM1 mutations in 17%. These mutation rates are comparable to those reported in other studies focusing on the AYA demographic, where FLT3-ITD mutations are found in approximately 20-30% of cases, and NPM1 mutations in about 15-25%. Of note, the mutation rates in pediatric and young adult AML patients are similar to those observed in adult AML patients. These genetic markers are crucial for prognosis and treatment decisions, underscoring the importance of molecular profiling in this age group.^3,13,18^

Unlike AYA patients with ALL, who often benefit from pediatric-inspired treatment protocols and, generally, have a more favorable prognosis, those with AML face a poorer outlook. This is due to the higher prevalence of adverse cytogenetic features, such as FLT3-ITD mutations and complex karyotypes, and a lack of clinical trials tailored specifically for this age group. Thus, most AML treatment regimens for AYA patients are derived from pediatric or adult studies.^3,5,18^ Treatment regimens for AML in AYA and adult patients share similarities in their overall structure, typically involving intensive induction with cytarabine and anthracyclines, followed by post-remission phases. However, key differences emerge in the management approach and outcomes between these groups. AYA patients often receive treatment protocols that bridge pediatric and adult regimens, incorporating elements from both. The use of allogeneic hematopoietic stem cell transplantation (HSCT) as an intensification strategy varies between AYA and adult populations, with differing indications and rates based on factors such as country, study group, and whether the provider is pediatric or adult focused.^17^ Notably, while outcomes for AYA patients receiving HSCT have improved over time, their overall survival rates remain intermediate between those of children and older adults. Additionally, although transplant-related mortality (TRM) has decreased across all age groups, it remains significantly higher in AYA patients compared to children, underscoring the need for age-specific considerations in treatment planning and supportive care for this unique patient population.^19^

In our retrospective study, we observed that non-AYA (adult) patients who underwent transplants experienced significantly better OS and PFS compared to those who did not receive transplants. This aligns with existing evidence that transplantation can enhance outcomes for adult patients, though it is typically an option only for those who are healthy enough to handle the procedure. Interestingly, for adolescents and young adults (AYA), there was no significant difference in OS or PFS between those who received transplants and those who did not. This might indicate that the benefits of transplantation are less pronounced in AYAs, or it could mean that other factors, like the effectiveness of initial chemotherapy, are more influential in this age group. These findings underscore the differences in treatment and outcomes between AYA and non-AYA AML patients. While AYAs often receive more intensive treatments and have higher rates of transplantation, the impact on survival can vary. This highlights the importance of developing personalized treatment strategies that consider the unique characteristics and risk profiles of each patient.

During chemotherapy, AYA patients are particularly prone to treatment-related complications, such as nausea and vomiting, which can significantly impact their quality of life and adherence to treatment regimens. Managing these side effects effectively is crucial, as they can lead to malnutrition, dehydration, and a decreased ability to tolerate further chemotherapy cycles.^20^ In addition to physical side effects, AYA patients face substantial psychosocial challenges. This age group is at a critical stage of developing autonomy and establishing their identity, which can be disrupted by the demands of cancer treatment. Issues related to social interactions, sexuality, education and career development are particularly pronounced, as treatment often requires extended hospital stays and can interfere with normal life progression.^21^

Furthermore, the risk of secondary malignancies due to chemotherapy or HSCT is a significant concern, with incidence rates reported as high as 1.5%. This risk necessitates careful long-term monitoring and follow-up care to detect and manage any subsequent cancers early.^22,23^ The potential for late effects of treatment, such as infertility, cardiotoxicity, and neurocognitive deficits, also requires attention. These factors underscore the importance of a multidisciplinary approach to care, which includes not only oncologists but also psychologists, social workers and other specialists who can address the comprehensive needs of AYA patients. Despite its valuable insights into AML characteristics and outcomes in AYA and non-AYA populations at a tertiary hematology center in Qatar, there are important limitations to our study. The single-center design and demographic composition, predominantly male patients of Asian descent, may limit the generalizability of our findings to broader populations. The retrospective nature introduces potential selection and information biases, and the 24-month median follow-up period may not fully capture late events, which are particularly relevant in AML. Although we collected detailed clinical and cytogenetic data for both age groups, our analysis did not control for potential confounding variables such as comorbidities and socioeconomic factors, which could differentially impact treatment decisions and survival outcomes between AYA and older patients. A significant limitation was the lack of standardized minimal residual disease (MRD) assessment, which is now recognized as a crucial prognostic tool for risk stratification and treatment decision-making in AML. While initial genetic analysis focused on key mutations like FLT3-ITD and NPM1, this limitation has been subsequently addressed through the implementation of comprehensive next generation sequencing (NGS) myeloid testing covering 68 mutations at our center. Notable gaps in our study include the absence of quality-of-life assessments and psychosocial impact analyses, which likely differ significantly between age groups. Treatment heterogeneity, including variations in FLAG-Ida protocols and stem cell transplantation criteria across age groups, may have influenced our comparative results. These limitations inform opportunities for future research, including multi-center studies with diverse patient populations, prospective designs with longer follow-up periods, integration of comprehensive molecular profiling and MRD monitoring, assessment of quality-of-life outcomes, and standardization of treatment protocols, which would strengthen our understanding of age-specific AML characteristics and improve the applicability of findings across both AYA and adult populations.

## Conclusion

Our comparative analysis of AML between AYA and non-AYA populations at our center reveals critical insights into age-specific characteristics and outcomes. Despite AML being traditionally associated with older adults, Qatar’s demographic structure provides a unique opportunity to study both age groups, with a significant number of cases among AYAs (aged 15-39). Our research demonstrates distinct cytogenetic risk profiles between age groups, with AYA patients showing different patterns of genetic mutations such as FLT3-ITD and NPM1 compared to adults, underscoring the need for age-specific molecular profiling to guide personalized treatment. While AYA patients often receive treatment protocols that blend pediatric and adult regimens, older adults typically follow standard adult protocols. Our findings show that although HSCT significantly improves outcomes in non-AYA patients, its benefits in AYAs are less pronounced, suggesting that initial chemotherapy effectiveness may play a more crucial role in younger patients. Treatment-related complications and toxicity profiles differ between age groups, with AYA patients demonstrating better tolerance but facing unique psychosocial challenges that impact their quality of life and treatment adherence. The non-AYA group, while more susceptible to treatment-related complications, shows different patterns of disease resistance and relapse. These age-specific differences necessitate tailored multidisciplinary approaches to care. Addressing these distinct needs through personalized treatment plans, increased clinical trial participation, and comprehensive supportive care is essential to optimize outcomes for both age groups. Our findings emphasize the importance of age-adapted strategies in AML management, potentially leading to improved survival rates and quality of life across all age groups

### Conflict of interest

On behalf of all authors, the corresponding author states that there is no conflict of Interest.

### Statement of Ethics

This study protocol was reviewed and approved by Hamad Medical Corporation Medical Research Center. Patient consent was not required as this study was based on publicly available data.

### Authorship Contributions

Conception and design of the study: S. Fareed, A. Al-Mashdali

Methodology: S. Fareed, D. Soliman

Validation: S. Fareed, D. Soliman, A. Al-Mashdali,

Acquisition of data: A. Al-Mashdali, Y. Mulikandathil, A. Alshurafa, S. Aldali, D. Mudawi, A. Yousef, H. Shwaylia, M. Abdulgayoom

Statistical analysis: K. Singh, Drafting the manuscript: S. Fareed, A. Al-Mashdali

Revising the manuscript for intellectual content: S. Fareed, A. Al-Mashdali, F. Abbas, A. Hamad

Approval of the version of the manuscript to be published: All authors have read and agreed to the published version of the manuscript

Supervision: H. Cherif, M. Yassin

## Data Availability

All data generated or analyzed during this study are included in this article. Further enquiries can be directed to the corresponding author.

## References

[1] Döhner H., Wei A. H., Appelbaum F. R., Craddock C., DiNardo C. D., Dombret H.. (2022). Diagnosis and management of AML in adults: 2022 recommendations from an international expert panel on behalf of the ELN. Blood.

[2] Creutzig U., Kutny M. A., Barr R., Schlenk R. F., Ribeiro R. C. (2018). Acute myelogenous leukemia in adolescents and young adults. Pediatr Blood Cancer.

[3] Lalayanni C., Demosthenous C., Iskas M., Kelaidi C., Papathanasiou M., Syrigou A.. (2022). Adolescents and young adults (AYA) with acute myeloid leukemia (AML): real-world long-term results and age-specific outcomes. Leuk Lymphoma.

[4] Chen R., AlHumaid M., Daher-Reyes G., Atenafu E. G., Chan S., Gupta V.. (2024). Outcome of adolescents and young adult acute myeloid leukemia patients compared with middle-aged patients: A single centre retrospective experience. Leukemia Research.

[5] Ofran Y., Rowe J. M. (2014). Acute myeloid leukemia in adolescents and young adults: challenging aspects. Acta Haematol.

[6] Abrahamsson J., Forestier E., Heldrup J., Jahnukainen K., Jónsson O. G., Lausen B.. (2011). Response-guided induction therapy in pediatric acute myeloid leukemia with excellent remission rate. J Clin Oncol.

[7] Creutzig U., Büchner T., Sauerland M. C., Zimmermann M., Reinhardt D., Döhner H.. (2008). Significance of age in acute myeloid leukemia patients younger than 30 years: a common analysis of the pediatric trials AML-BFM 93/98 and the adult trials AMLCG 92/99 and AMLSG HD93/98A. Cancer.

[8] Gamis A. S., Alonzo T. A., Meshinchi S., Sung L., Gerbing R. B., Raimondi S. C.. (2014). Gemtuzumab ozogamicin in children and adolescents with de novo acute myeloid leukemia improves event-free survival by reducing relapse risk: results from the randomized phase III Children’s Oncology Group trial AAML0531. J Clin Oncol.

[9] Creutzig U., Zimmermann M., Bourquin J.-P., Dworzak M. N., Fleischhack G., Graf N.. (2013). Randomized trial comparing liposomal daunorubicin with idarubicin as induction for pediatric acute myeloid leukemia: results from Study AML-BFM 2004. Blood.

[10] Tsukimoto I., Tawa A., Horibe K., Tabuchi K., Kigasawa H., Tsuchida M.. (2009). Risk-stratified therapy and the intensive use of cytarabine improves the outcome in childhood acute myeloid leukemia: the AML99 trial from the Japanese Childhood AML Cooperative Study Group. J Clin Oncol.

[11] Gibson B. E. S., Webb D. K. H., Howman A. J., De Graaf S. S. N., Harrison C. J., Wheatley K.. (2011). Results of a randomized trial in children with Acute Myeloid Leukaemia: medical research council AML12 trial. Br J Haematol.

[12] Juliusson G., Antunovic P., Derolf A., Lehmann S., Möllgård L., Stockelberg D.. (2009). Age and acute myeloid leukemia: real world data on decision to treat and outcomes from the Swedish Acute Leukemia Registry. Blood.

[13] Pemmaraju N., Kantarjian H., Ravandi F., Nogueras-Gonzalez G. M., Huang X., O’Brien S.. (2016). Patient Characteristics and Outcomes in Adolescents and Young Adults (AYA) With Acute Myeloid Leukemia (AML). Clin Lymphoma Myeloma Leuk.

[14] Trama A., Botta L., Foschi R., Ferrari A., Stiller C., Desandes E.. (2016). Survival of European adolescents and young adults diagnosed with cancer in 2000-07: population-based data from EUROCARE-5. Lancet Oncol.

[15] Woods W. G., Franklin A. R. K., Alonzo T. A., Gerbing R. B., Donohue K. A., Othus M.. (2013). Outcome of adolescents and young adults with acute myeloid leukemia treated on COG trials compared to CALGB and SWOG trials. Cancer.

[16] Durani U., Go R. S. (2017). Racial and ethnic disparities in the survival of adolescents and young adults with acute myeloid leukemia: a retrospective study using the US National Cancer Data Base. Leuk Lymphoma.

[17] O’Dwyer K., Freyer D. R., Horan J. T. (2018). Treatment strategies for adolescent and young adult patients with acute myeloid leukemia. Blood.

[18] Creutzig U., Kutny M. A., Barr R., Schlenk R. F., Ribeiro R. C. (2018). Acute myelogenous leukemia in adolescents and young adults. Pediatr Blood Cancer.

[19] Majhail N. S., Brazauskas R., Hassebroek A., Bredeson C. N., Hahn T., Hale G. A.. (2012). Outcomes of allogeneic hematopoietic cell transplantation for adolescent and young adults compared with children and older adults with acute myeloid leukemia. Biol Blood Marrow Transplant.

[20] Bhatia S., Landier W., Shangguan M., Hageman L., Schaible A. N., Carter A. R.. (2012). Nonadherence to oral mercaptopurine and risk of relapse in Hispanic and non-Hispanic white children with acute lymphoblastic leukemia: a report from the children’s oncology group. J Clin Oncol.

[21] Penson R. T., Rauch P. K., McAfee S. L., Cashavelly B. J., Clair-Hayes K., Dahlin C.. (2002). Between parent and child: negotiating cancer treatment in adolescents. Oncologist.

[22] Leiper A. D. (2002). Non-endocrine late complications of bone marrow transplantation in childhood: part II. Br J Haematol.

[23] Sun C.-L., Francisco L., Kawashima T., Leisenring W., Robison L. L., Baker K. S.. (2010). Prevalence and predictors of chronic health conditions after hematopoietic cell transplantation: a report from the Bone Marrow Transplant Survivor Study. Blood.

